# ﻿A honey bee fossil (Hymenoptera, Apidae) from the Late Pliocene to Early Pleistocene Teragi Group, Hyogo Prefecture, Japan: Bridging a gap in *Apis* evolutionary history

**DOI:** 10.3897/zookeys.1255.162389

**Published:** 2025-10-13

**Authors:** Yui Takahashi, Jun-ichi Takahashi

**Affiliations:** 1 Keio Yochisha Elementary School, 2-35-1, Shibuya-ku, Tokyo 150-0013, Japan Keio Yochisha Elementary School Tokyo Japan; 2 Faculty of Life Sciences, Kyoto Sangyo University, Kamigamo, Kyoto 603-8555, Japan Kyoto Sangyo University Kyoto Japan

**Keywords:** Apini, Apoidea, Cenozoic, fossil record, insect fossil, lacustrine deposit

## Abstract

A new fossil honey bee Apis (Apis) aibai**sp. nov.** was discovered in the Late Pliocene–Early Pleistocene lacustrine deposit in Hyogo Prefecture, Japan. Fossil species are identified based on their distinct forewing venation, thick, lighter-colored abdomens, and hind legs. Honeybee fossils exhibit a highly uneven distribution across time. Fossil species are primarily derived from older Oligocene–Miocene deposits, mostly in Europe and China, while fossils of a few modern species have been recovered from younger Pleistocene and Holocene deposits. Apis (Apis) aibai**sp. nov.** bridges the gap between older and younger fossil records. Additionally, this species represents the most recent extinct honey bee and the oldest known record of the subgenus Apis.

## ﻿Introduction

The honey bee, a general term for hymenopteran insects of the genus *Apis* (family Apidae), plays a vital role as a pollinator for both natural and agricultural ecosystems (e.g., Partap 2011). In addition, these insects maintain some of the closest ties to human society (e.g., Oldroyd and Wongsiri 2009). There are at least nine existing honey bee species and three subgenera from the genus *Apis*: *A. florea* Fabricius and *A. andreniformis* Smith of dwarf honey bees (subgenus Micrapis Ashmead); *A. dorsata* Fabricius and *A. laboriosa* Smith of giant honey bees (subgenus Megaapis Ashmead); and *A. mellifera* Linnaeus, *A. koschevnikovi* Enderlein, *A. cerana* Fabricius, *A. nuluensis* Tingek et al., and *A. nigrocincta* Smith of cavity-nesting bees (Apis s. str., subgenus Apis Linnaeus) (Raffiudin and Crozier 2007; Oldroyd and Wongsiri 2009; Lo et al. 2010; Han et al. 2012). The native range of *A. mellifera* covers Europe, Africa, the Middle East, Central Asia, and western China, whereas the other eight congeners are primarily found in Asia (Ji 2021). The distributions of the eight Asian species overlap in the tropical and subtropical zones of South and Southeast Asia, whereas only the range of *A. cerana* extends northward to the temperate zone (e.g., Hepburn and Radloff 2011).

The fossil record of *Apis* is disproportionately derived from Eocene–Miocene deposits in Europe and China, with only a few exceptions from the Pleistocene age (summarized in Engel 1998; Kotthoff et al. 2011). Based on specimens derived from these localities, Engel (1999) reclassified fossil examples of *Apis* and categorized them into six species: *A. armbrusteri* Zeuner of the fossil subgenus †*Cascapis* Engel, *A. vetusta* Engel of subgenus †*Priorapis* Engel, *A. henshawi* Cockerell, *A. longtibia* Zhang, *A. miocenica* Hong, and *A. petrefacta* (Říha) of subgenus †*Synapis* Cockerell. Since then, only a few fossil findings were discovered, such as Apis (Megapis) lithohermaea Engel from the Middle Miocene deposits of Iki Island, Japan (Engel 2006), Apis (Cascapis) nearctica Engel, Hinojosa-Díaz, and Rasnitsyn from the Middle Miocene deposits of west-central Nevada, USA (Engel et al. 2009), and Apis (Synapis) dalica Engel and Wappler from the Middle Miocene deposits distributed in southeastern Yunnan Province, China (Engel et al. 2018). These records reinforce the bias toward Eocene–Miocene materials. This study discusses a fossil honey bee discovered from a previously undocumented interval in the fossil record.

## ﻿Material and methods

**Figure 1.**
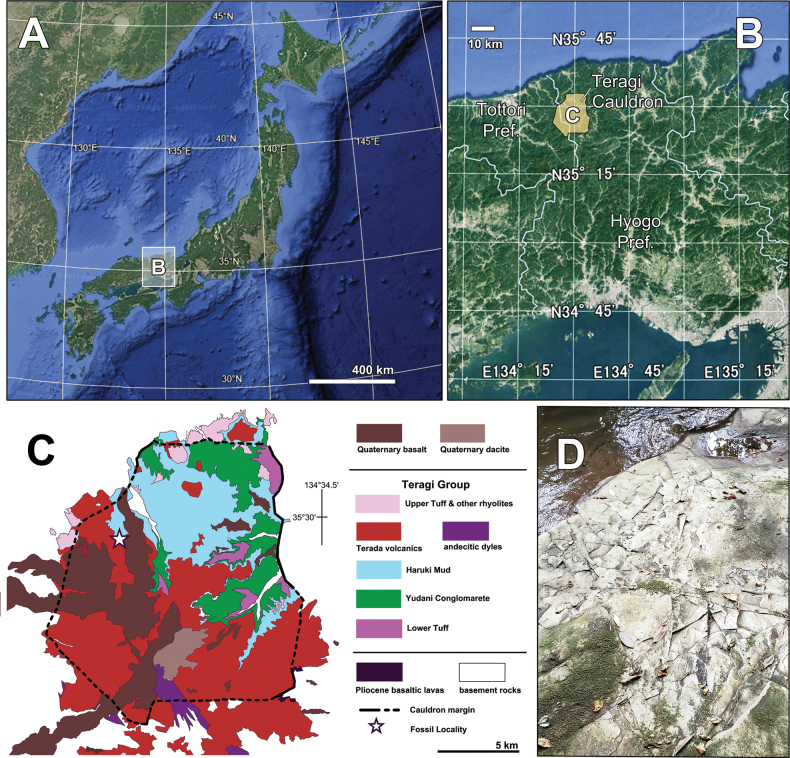
Locality of the studied specimen. A. Map of the Japanese island; B. Distribution of the Teragi Cauldron between Tottori and Hyogo prefectures. The basemap of A, B. are digital map images exported from Google Earth Pro 2025; C. Simplified geological map of the Teragi Cauldron based on Furuyama and Nagao (2004), representing the fossil locality; D. Photo of the riverside fossil locality (35°29'39.41"N, 134°26'24.53"E), with whitish tuffaceous siltstones (photo provided by K. Nakai).

Fossil specimens were collected from the lacustrine deposits of the Teragi Group. The group (ca. 3.1–2.2 Ma: Late Pliocene–Early Pleistocene) is distributed across eastern Tottori and northwestern Hyogo prefectures, southwestern Japan (Fig. 1), comprising volcanic rocks, pyroclastic frows, and clastic sedimentary rocks, which fill a volcanic collapse basin called the Teragi Cauldron. The Teragi Group is stratigraphically composed of the Lower Tuff, Yudani Conglomarete, The Haruki Mudstone (generally called Haruki Mud), Terada volcanics, and Upper Tuff and other rhyolites almost in ascending order (Furuyama and Nagao 2004). However, the stratigraphy is not simply due to the interfingering of the Haruki Mud, Terada volcanics, Upper Tuff and other rhyolites. The age of the group was determined via K–Ar radiometric dating from volcanic rocks as 3.13±0.08 Ma from Lower Tuff and 2.28 ± 0.07 Ma from Upper Tuff and other rhyolites (Furuyama and Nagao 2004).

The Haruki Mud (ca. 2.8–2.2 Ma: Late Pliocene–Early Pleistocene), fossiliferous lacustrine deposits characterized by thin alternating beds of sandstone and siltstone interbedded with numerous layers of tuff. The lower age was determined using K–Ar radiometric dating from the Terada volcanics (Furuyama and Nagao 2004). The Haruki Mud is exposed mainly northeast of the cauldron, and the main fossil site is Umigami, Shin’onsen Town, Hyogo Prefecture. Many fossil leaves and insects have also been found in this locality (Inoue 1986). However, fossil fauna and flora are not well understood, owing to a lack of detailed taxonomic studies. In Inoue (1986), Dr Uemura personally commented that fossil plants are mainly composed of leaves belonging to the families Aceraceae, Betulaceae, Fagaceae, and Ulmaceae and that the paleoenvironment of the area seemed to be a temperate forest. Kinugasa et al. (1968) identified five insect fossils and described two specimens as Asilidae gen. et sp. indet. and *Camponotus* sp. Furthermore, Arita and Yamana (1970) described a spider fossil as Philodromidae gen. et sp. indet. Kinugasa (1981) described five genera and eight species of Ephemeroptera as modern species; however, given their geological age, this identification is debatable (personal comment). Fujiyama (1982) described two cicadids *Graptopsaltria* sp. and *Meimuna* sp. Inoue (1986) listed 75 species belonging to 11 orders based on photographs; however, no taxonomic descriptions were provided. As our knowledge of fossil fauna and flora is limited, the reclassification and re-evaluation of fossils from Haruki Mud have begun. Recently, Aiba et al. (2025) described a new fossil species of the nymphalid butterfly, *Tacola kamitanii* Aiba et al.

The studied fossil is preserved in the whitish tuffaceous siltstone of Haruki Mud (Fig. 1D) and deposited in the Museum of Unique Insect Fossils, managed by the Shin’onsen Town Board of Education, Shin’onsen Town, Hyogo Prefecture, with repository number SOU-002. The fossil was observed under a Leica M205 C microscope (Leica Corporation, Wetzlar, Germany). Images were captured using a Leica MC170HD microscope (Leica Application Suite version 4.1.3, Leica Corporation). The images were polished and their contrast and tonality were adjusted using Adobe Photoshop TM Version CS6 (Adobe Systems Incorporated, San Jose, CA, USA).

The nomenclature used in this study follows Engel (2001) and Tan et al. (2008). For comparison, we refer to modern specimens deposited at Kyoto Sangyo University without repository numbers.

## ﻿Systematic paleontology

### ﻿Order Hymenoptera Linnaeus, 1758

**Family Apidae Latreille, 1802**

**Genus *Apis* Linnaeus, 1758**

**subgenus Apis Linnaeus, 1758**

#### ﻿ Apis (Apis) aibai sp. nov.

Taxon classificationAnimaliaHymenopteraApidae

29DED547-14D8-57E9-8F46-611E0C8D4209

https://zoobank.org/EB5BB274-3AF3-426E-8805-B0DCC91FC954

**Figure 2.**
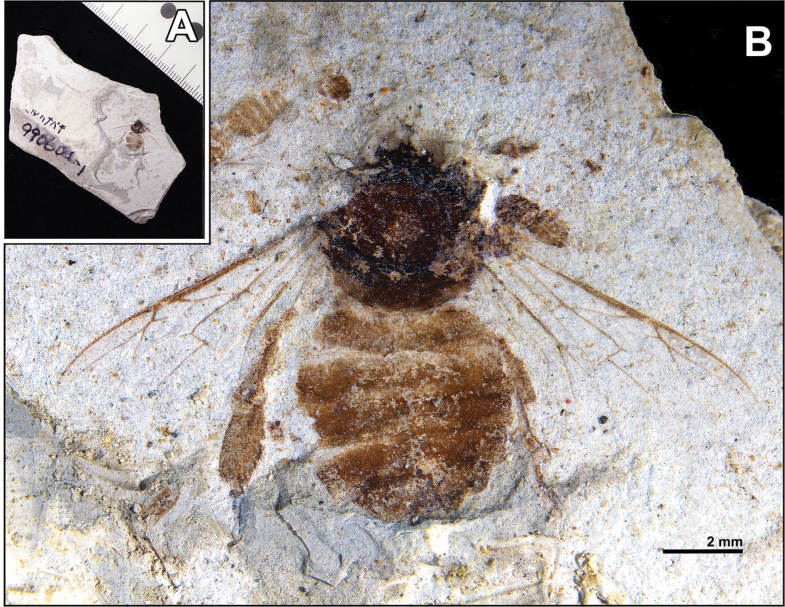
Photographs of Apis (Apis) aibai sp. nov. (SOU-002). A. Fossil-bearing piece (38.3 mm × 67.1 mm × 9.8 mm); B. Studied fossil reflecting dorsal view of SOU-002.

**Figure 3.**
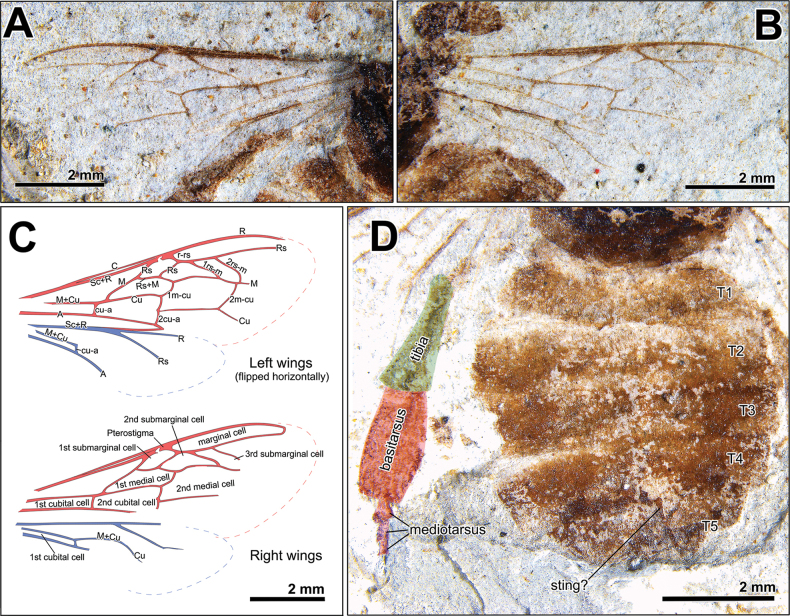
Photographs and illustrations of Apis (Apis) aibai sp. nov. (SOU-002). A. Left wings; B. Right wings; C. Line drawings of wings. The uppers are left wings and lowers right (left wings are flipped horizontally); D. Metasoma and hind legs.

Figs 2, 3 [New Japanese name: Tajima-mitsubachi]

##### Type material.

**Holotype.** • SOU-002 (Fig. 2A, B). **Worker/ Female.** An impressed/compressed individual in dorsal view. Head almost lacking. Mesosoma fine structures hard to interpret, with partly preserved obliquely outstretched forewings, hindwings, and hind legs. Metasoma outline and individual segments discernible. No counterpart. The holotype is deposited in the Museum of Unique Insect Fossils, Shin’onsen Town, Hyogo Prefecture.

##### Diagnosis.

Medium-sized honey bee (body length approximately 10.0 mm). Wings hyaline. Forewing length approximately 8.4 mm. Vein 1Rs strongly slanted posterobasally, as long as vein 1Rs+M, subparallel to vein 2Rs, forming a slender subparallelogram-shaped 1^st^ submarginal cell. Cross vein 1cu-a 0.7–0.8 times its length distant to vein 1M (basal vein). Cubital index 4.5. Hind tibia 2.3 times longer than wide, as long as basitarsus. Abdomen 1.4 times wider than thorax.

##### Description.

**Worker.** Total body length 9.81 mm (as preserved). Head almost lacking, black in color.

Mesosoma apparently darker colored than metasoma, 3.52 mm long, 3.94 mm wide, with rounded outline. Pronotum black colored, not well preserved. Scutum 2.04 mm long, 2.98 mm wide, both anterior and posterior margins rounded. Scutoscutellar groove deep. Scutellum black colored, 0.52 mm long, with rounded posterior margin. Propodeum 0.56 mm long, 3.47 mm wide, with rounded posterior margin.

Forewing hyaline with complete venation, 7.8–8.1 mm long (as preserved). Reconstructed length approximately 8.4 mm long, width 2.9 mm wide. Marginal cell elongated, 3.12 mm long, 0.36 mm wide, about 8.6 times as long as wide, scarcely tapering apically, bluntly rounded at apex. Pterostigma small, 0.32 mm long, not projecting beyond cross vein r-rs. Vein 1Rs straight, 0.54–0.57 mm long, strongly slanted posterobasally almost in line with vein 1M, subparallel to vein 2Rs. Vein 2Rs weakly sinuate, 0.59–0.64 mm long, as long as vein Rs+M. Three submarginal cells present. 1^st^ submarginal cell slender subparallelogram in shape, vein Rs+M 0.56–0.60 mm long. Cross vein r-rs 0.30–0.36 mm long, as long as anterior margin of 2^nd^ submarginal cell. 2^nd^ submarginal cell elongated, due to posterobasally slanted 2Rs and posteroapically slanted cross vein 1rs-m. Vein 2M straight, 0.54–0.56 mm long. Vein 3M anteriorly arched, 1.23–1.25 mm. Anterior margin of cell short, 0.26–0.30 mm long, one-third of cross vein 1rs-m. 3^rd^ submarginal cell relatively not large, subparallelogram in shape, anterior border not long, 0.61–0.67 mm long, posterior margin 0.59 mm long. Cross vein 1rs-m sinuate, 0.90–0.91 mm long, almost parallel to cross vein 2rs-m, meeting 2^nd^ medial cell in five-seventh of its upper base. Vein 4M posteriorly arched, 0.49 mm. Cross vein 2rs-m sinuate, 0.93 mm long, a without abscissal stub “aRs2”. 1^st^ medial cell subtrapezoid in shape, about 3.7–4.0 times as long as wide. Vein 1Cu 1.45–1.55 mm long. Cross vein 1m-cu 0.50–0.54 mm long, strongly arched outwardly at middle, joining 2^nd^ submarginal cell in one-fourth of its base. 2^nd^ medial cell large, subparallelogram in shape, about 2.2 times as long as wide. Vein 2Cu 0.40 mm. Vein 3Cu straight, 1.57 mm long. Cross vein 2m-cu 0.85 mm long, weakly arched outwardly, meeting 3^rd^ submarginal cell in about five-sixth of its base (vein 5M 0.11 mm long, cubital index about 4.5). Vein 1M (basal vein) 0.93–1.01 mm long, slightly sinuate. Cross vein 1cu-a 0.34–0.37 mm long, 0.27–0.28 mm distant to vein 1M, perpendicular to vein A. Vein 2Cu slightly slanted posterobasally, almost in line with cross vein 2cu-a. 2^nd^ cubital cell elongated, apically widest, 1.67–1.80 mm long, 0.77–0.81 mm wide. Cross vein 2cu-a 0.39–0.42 mm long, parallel to 1cu-a.

Hindwing hyaline, reconstructed length 5.8 mm long. Humeri not preserved. Vein Sc+R straight. Rs almost straight, forming an angle of approximately 30° with vein R. Distal abscissa of vein Rs present. Distal abscissa of vein M uncertain. Cross vein cu-a 0.20–0.23 mm long, slightly inclined posteroapically. Vein A parallel to vein 1M+Cu, forming elongated cubital cell. Jugal and vannal lobes uncertain.

Hind tibia not dark colored, widening apically, 2.01 mm long, 0.86 mm wide at most, dorsal margin slightly concave, ventral margin straight, without tibial spurs. Basitarsus enlarged and flattened, rectangular in shape, 1.92 mm long, 1.02 mm wide at middle, dorsal margin almost straight, ventral margin convex. 1^st^ mediotarsus largely widening apically, 0.40 mm long, 0.36 mm wide. 2^nd^ mediotarsus 0.25 mm long, 0.18 mm wide. 3^rd^ mediotarsus 0.25 mm long, 0.15 mm wide. Remaining portion poorly preserved.

Metasoma not dark colored, 5.15 mm long, 5.45 mm wide, with five terga (T1–T5) visible. Darker colored bands remained on posterior parts of T4 and T5. T1 widely transverse, 0.87 mm long, 4.49 mm wide. T2 widest, 1.17 mm long, 5.45 mm wide. T3 0.97 mm long. T4 0.66 mm long, 4.77 mm wide. T5 1.03 mm long, 4.09 mm wide. Sting? present at T5, may be displaced forward during fossilization.

##### Etymology.

The species is named to honor Hiroaki Aiba, a Japanese paleontologist who described a new fossil species of a nymphalid butterfly found at the same fossil site in Umigami (Aiba et al. 2025). He not only studied Japanese fossil insects but also contributed to Earth science education through the development of fossil collection activities.

##### Comparisons.

The forewing venation of the honey bees *Apis* is distinctive and can be easily recognized from the pattern and shape of the veins. For instance, the long marginal cell without apical tapering, the presence of three submarginal cells, and cross veins 1rs-m and 2rs-m strongly slanted posteroapically (e.g., Zeuner and Manning 1976; Engel 1999). In addition to these characteristics, the enlarged and flattened basitarsus proved that the studied material is a worker bee (Fig. 3C). The reconstructed forewing length of the fossil clearly distinguished the dwarf and giant honey bee subgenera *Megapis* and *Micrapis*. The fossil material also does not match with extinct subgenera as vein 1M (basal vein) of †*Synapis* is only slightly distad from cross vein 1cu-a, straight vein 1M of †*Priapis* is confluent with cross vein 1cu-a, and the 3^rd^ submarginal cell of †*Cascapis* is larger (Engel 2001). These facts demonstrate that the studied fossil is a member of the subgenus Apis.

As mentioned, five modern species comprise the subgenus, and we compared the fossil with them. The fossil clearly differs from *A. mellifera* and *A. koschevnikovi* because the cubital index of *A. mellifera* is much smaller and that of *A. koschevnikovi* is much larger than that of the new fossil (e.g., Hadisoesilo et al. 2008; Hassona 2017). The cubital index of *A. nuluensis* is also lower than that of the fossil specimen (Tingek et al. 1996). The studied fossil resembles *A. cerana*, but the 1^st^ submarginal cell of the latter is not as slender as the fossil material (Tan et al. 2008). Additionally, subspecies *Apis cerana indica* Fabricius has a longer hind tibia than that of the studied specimen (Mattu and Verma 1984). Furthermore, in Japan, there is only one native honey bee subspecies, *A. cerana japonica* Radoszkowski, most of which have a forewing abscissal stub “aRs2” (Tan et al. 2008). The forewing venation of the new fossil closely resembles that of *A. nigrocincta*, as vein 1Rs is strongly slanted posterobasally, almost in line with vein 1M, which forms the slender 1^st^ submarginal cell (Fig. 3A–C). However, cross vein 1cu-a is more strongly distad to vein 1M in the latter, because the distance between vein 1cu-a and vein M is shorter than the length of vein 1cu-a itself, but that of *A. nigrocincta* is the same length or slightly longer.

## ﻿Discussion

First, this discovery fills the fossil age gap. The honeybee fossil record is notably uneven, with most Oligocene–Miocene specimens originating from Europe and China (e.g., Hong 1983; Zhang 1989; Engel 1999; Nel et al. 1999). A few younger records are occupied by the Pleistocene and Holocene representatives, which are identified as modern species. Several *Apis mellifera* individuals were included in the Eastern African copals (Foord 1890; Cockerell 1909); these copals are from the Late Pleistocene or even younger (Burleigh and Whalley 1983). A petrified comb of *A. cerana* was reported from the Late Cenozoic (detailed age uncertain) cave deposit of Kuala Lumpur, Malaysia (Stauffer 1979). Therefore, A. (A.) aibai sp. nov. of the Late Pliocene–Early Pleistocene age is direct evidence connecting these older and younger fossil records and is assigned as the youngest extinct species.

Second, the presence of A. (A.) aibai sp. nov. fills the gap between the estimated evolutionary tree and fossil records. Documentation of the subgenus Apis is limited to the Pleistocene, as old as above, and therefore, the new fossil species is the oldest representative belonging to the subgenus. The material discussed here provides evidence for the existence of *Apis* s. str. dating back to the Late Pliocene–Early Pleistocene at the eastern end of Asia. A maximum likelihood tree, recently reconstructed based on mitochondrial genome sequence data, suggests that the subgenus might have appeared in the Late Miocene (Ji 2021); however, there have been no Pliocene fossil data for the subgenus until today. Thus, Apis (Apis) aibai sp. nov. contributes to bridging the gap between fossil studies and genomic estimations.

Finally, the forewing venation of A. (A.) aibai sp. nov. explicitly resembles *A. nigrocincta*, which is only distributed in the modern Philippines and Indonesia, in terms of certain characteristics, such as vein 1Rs strongly slanted posterobasally, as long as vein 1Rs+M, subparallel to vein 2Rs, forming a slender subparallelogram-shaped 1^st^ submarginal cell. This suggests that the *A. nigrocincta*-related extinct group, including the new species, historically had a wider distribution, ranging to temperate Japan of the Late Pliocene–Early Pleistocene age. An interesting hypothesis is that the *A. nigrocincta*-related extinct group may be the ancestral bees of *A. cerana*, which is the only native honey bee species in Japan. In fact, maximum likelihood trees based on the mitochondrial genome indicate that *A. nigrocincta* and *A. cerana* have an intimate relationship (Lo et al. 2010; Takahashi et al. 2018) and that their common ancestral lineage may have appeared from the Pliocene (Ji 2021). We may have obtained information regarding the ancestral lineage of the new specimen. Thus, the discovery of a new fossil species, A. (A.) aibai sp. nov., has clarified the gap between fossils and recent honey bees.

## Supplementary Material

XML Treatment for Apis (Apis) aibai

## ﻿Additional information

### Use of AI

No use of AI was reported.

### Author ORCIDs

Yui Takahashi https://orcid.org/0000-0003-2861-9940

Jun-ichi Takahashi https://orcid.org/0000-0001-6180-0501

### Data availability

All of the data that support the findings of this study are available in the main text.
